# Evolutionary history and stress regulation of the lectin superfamily in higher plants

**DOI:** 10.1186/1471-2148-10-79

**Published:** 2010-03-18

**Authors:** Shu-Ye Jiang, Zhigang Ma, Srinivasan Ramachandran

**Affiliations:** 1 Temasek Life Sciences Laboratory, 1 Research Link, the National University of Singapore, Singapore 117604

## Abstract

**Background:**

Lectins are a class of carbohydrate-binding proteins. They play roles in various biological processes. However, little is known about their evolutionary history and their functions in plant stress regulation. The availability of full genome sequences from various plant species makes it possible to perform a whole-genome exploration for further understanding their biological functions.

**Results:**

Higher plant genomes encode large numbers of lectin proteins. Based on their domain structures and phylogenetic analyses, a new classification system has been proposed. In this system, 12 different families have been classified and four of them consist of recently identified plant lectin members. Further analyses show that some of lectin families exhibit species-specific expansion and rapid birth-and-death evolution. Tandem and segmental duplications have been regarded as the major mechanisms to drive lectin expansion although retrogenes also significantly contributed to the birth of new lectin genes in soybean and rice. Evidence shows that lectin genes have been involved in biotic/abiotic stress regulations and tandem/segmental duplications may be regarded as drivers for plants to adapt various environmental stresses through duplication followed by expression divergence. Each member of this gene superfamily may play specialized roles in a specific stress condition and function as a regulator of various environmental factors such as cold, drought and high salinity as well as biotic stresses.

**Conclusions:**

Our studies provide a new outline of the plant lectin gene superfamily and advance the understanding of plant lectin genes in lineage-specific expansion and their functions in biotic/abiotic stress-related developmental processes.

## Background

Lectins are carbohydrate-binding proteins that specifically recognize diverse sugar structures and mediate a variety of biological processes [1,2]. Lectin proteins contain at least one carbohydrate-binding domain. Based on this, three major types of lectins are distinguished, namely merolectins, hololectins and chimerolectins [3]. The merolectins have only single carbohydrate-binding domain and the hololectins contain two or more domains which are either identical or very homologous. The chimerolectins are fusion proteins consisting of one or more carbohydrate-binding domains and unrelated domains. Lectins are ubiquitous in nature, found in all kinds of organisms, from virus to humans [4]. Plant lectins are usually considered as a very heterogeneous group of proteins because comparative biochemical studies clearly indicate that they differ from each other with respect to their biochemical/physicochemical properties, molecular structure, carbohydrate-binding specificity and biological activities [5]. Therefore, it is difficult to find a widely acceptable way to classify plant lectins. Currently, several attempts have been made to group plant lectins. One of them was based on the carbohydrate-binding specificity. As a result, mannose- mannose/glucose-, mannose/maltose-, Gal/GalNAc, GlcNAc/(GlcNAc)_n_-, fucose- and salic acid-binding lectins have been distinguished ([5,6]. This classification emphasizes the use of lectins as tools with different carbohydrate-binding specificity. However, evolutionarily unrelated lectins may also be classified together. Besides this, another classification has been suggested, which is based on 3D structures of lectins. They classified lectins as 6 major groups including α-D-mannose-specific plant lectin (monocot lectin), agglutinin with hevein domain, β-prism plant lectin, β-trefoil lectin, cyanovirin-N homolog and legume lectin http://www.cermav.cnrs.fr/lectines/. Besides these, a more complicated classification system has been proposed which is based on either serological relationships or sequence similarities or both as well as their evolutionary relationships. Based on such criterion, 7 lectin families have been classified including the legume lectins, the monocot mannose-binding lectins, the chitin-binding lectins, the type 2 RIP and related lectins, jacalin-related lectins, Amaranthin lectins and Cucurbitaceae phloem lectins [6]. Recently, these authors made an update of the system since many new plant lectins have been isolated and characterized [7]. They have classified plant lectins into 12 families and at least one member in each family has been characterized in some detail and we named this system as "system 1". These families are as follows: ABA (*Agaricus bisporus *agglutinin), Amaranthin, CRA (chitinase-related agglutinin), Cyanovirin, EEA (*Euonymus europaeus *agglutinin), GNA (*Galanthus nivalis *agglutinin), Hevein, Jacalins, Legume lectin, LysM (lysin motif), Nictaba and Ricin_B families. However, the classification system was based on the available plant lectin information and animal lectins were not used to explore lectin-like members in the plant genomes.

Rapid progress has been achieved in genome sequencing with the great achievement in new sequencing technologies [8,9]. Both *Arabidopsis *and rice genomes have been completely sequenced [10-13] as model plants for dicot and monocot plants, respectively. Currently, soybean genome has also been completely sequenced (Soybean Genome Project, DoE Joint Genome Institute, http://www.phytozome.net/soybean). Up to now, genomes from 110 eukaryotes, 844 bacterial and 63 archaeal have been completely sequenced and published and the sequencing for 1028 eukaryotes, 2606 bacterial and 96 archaeal genomes are in progress based on the genome online database V 2.0 ([14]; http://www.genomesonline.org/gold.cgi, June 8, 2009). All these data provide us additional information to further analyze the lectin superfamily in their molecular evolution, classification and biological functions. However, limited data has been reported on the genome-wide characterization and molecular evolution of this superfamily in plants. As a result, little is known on the outline of all lectin genes in a completely sequenced plant genome and no new classification system has been proposed on the basis of the whole genome sequence information.

On the other hand, another major question that one may concern is on the biological functions of plant lectins. Many reviews or books have summarized the possible functions of plant lectins [4-6,15-18]. However, no definitive answers have been given. Generally, plant lectins have both internal and external activities [17]. The former refers to the functions acting within the plants during various developmental processes such as interactions with storage proteins or enzymes [17]. The latter includes the roles of lectins in response to various biotic and abiotic stresses. Evidence has shown the insecticidal activity of lectins against a spectrum of insects [19-24]. Some of lectin genes could be used for improving plant tolerance/resistance to various insects by transgenic technology [22,25-33]. However, such a desired effect in crop protection was not observed in some cases [34-36]. The protection functions of lectins against other biotic stresses were also reported including fungi [37-42] and virus [43,44]. Plant lectins are not only assumed to be part of the defense system [3,45], they have been also implicated as playing an important role in mediating recognition and specificity in the symbiosis with root nodule bacteria [46-49]. Besides biotic stresses, plant lectins may also play roles in abiotic stresses [50]. Reports have shown that several lectin genes exhibit differential expression abundance under various abiotic stresses including temperature shock, drought and high salinity stresses [51-56].

Since limited data is available on the genome-wide analysis of lectin genes, we do not know how many members of this family in a genome are involved in biotic and abiotic stress-related biological processes and how these genes have been expanded or evolved with such functions. In this report, we first identified and characterized all lectin genes encoded by the soybean, rice and *Arabidopsis *genomes. We then proposed a new classification system on the basis of protein domain structures and phylogenetic analyses. We also evaluated their expansion mechanisms and evolutionary history by investigating their duplication and/or transposition history. Subsequently, we examined their expression by full-length cDNA, Expression Sequence Tag (EST), microarray and Massively Parallel Signature Sequencing (MPSS [57]) datasets. Finally, we investigated their expression divergence under various stresses to further annotate their biological functions. Our analyses advance the understanding of plant lectin genes as being involved in lineage-specific expansion, and that they function in biotic and abiotic stress-related developmental processes. We also identified putative new lectin genes which were not be experimentally detected at present in plant genomes and provided a new outline of the plant lectin gene superfamily.

## Results and Discussion

### Genome-wide identification of the lectin superfamily in soybean, rice and *Arabidopsis*

**Figure 1:**
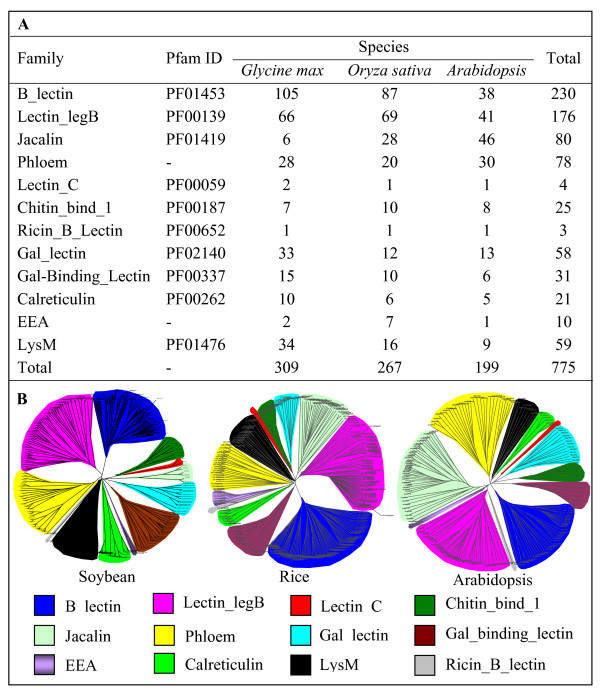
**Genome-wide identification of lectin superfamily members and their classification**. (A) All lectin members from 12 different lectin family in soybean, rice and *Arabidopsis *genomes. (B) Phylogenetic analysis of all lectin members in three different genomes.

To survey lectin genes in legume plants, the soybean (*Glycine max*) genome was selected as it has been completely sequenced. To better understand their expansion history and expression divergence, two other genomes were also selected for comparative analyses including rice (model plant for monocot) and *Arabidopsis *(model plant for dicot) genomes. We have used both BLAST and Hidden Markov model (HMM) searches (Methods) to identify lectin members presented in these genomes. After multiple cycles of searches, total of 349, 339 and 204 putative lectin genes have been detected in soybean, rice and *Arabidopsis*, respectively. These members were then subjected to the Pfam ([58]; http://pfam.sanger.ac.uk/) and SMART ([59]; http://smart.embl-heidelberg.de/) databases to confirm the presence of corresponding domains. The analysis revealed some members with incomplete domain structures, which have been confirmed by both domain searches and manual check. These members contain no typical domain structure and have no expression evidence with the characters of pseudogenes. Due to the low feasibility of phylogenetic analyses by integrating these partial fragments, we removed these members from our analyses although we may under-estimate the rate of gene duplication. Thus, our analyses reveal that soybean, rice and *Arabidopsis *genomes encode total of 309, 267 and 199 members of lectin superfamilies (Figure 1A). Their locus name, physical position and annotated protein sequences were deposited in the Additional file 1 (soybean), 2 (rice) and 3 (*Arabidopsis*).

### A new classification system of the lectin superfamily in higher plants

To classify these members, domain sequences from all members in each genome were aligned together and were then submitted to phylogenetic tree construction (Methods). The analyses show that all 3 organisms have 12 families of lectins (Figure 1B). They are named according to their domain description in the Pfam database, i.e., the B_lectin, Lectin_legB, Jacalin, Phloem, Lectin_C, Chitin_bind_1, Ricin_B_Lectin, Gal_lectin, Gal_binding_Lectin, Calreticulin, EEA and LysM families. In most cases, each family has a Pfam domain ID as shown in Figure 1A. However, no domain ID has been detected for the phloem and EEA families. The phloem domain was first structurally identified by Dinant et al. (2003) [60] with 4 conserved motifis and is characterized by a high frequency of charged residues and seven conserved Trp residues although phloem lectins were described in the seventies. Similarly, the EUL (Euonymus lectin) domain in the EEA family was recently identified [7,61] although the family members were described more than 20 years ago [62]. Among the total 12 identified families, both B_lectin and Lectin_legB are the largest families for soybean and rice. However, in *Arabidopsis*, the largest one is the Jacalin family followed by Lectin_legB, B_lectin and Phloem families (Figure 1A). The smallest families contain only one or two members including Lectin_C, Ricin_B_Lectin and EEA families (Figure 1).

**Figure 2:**
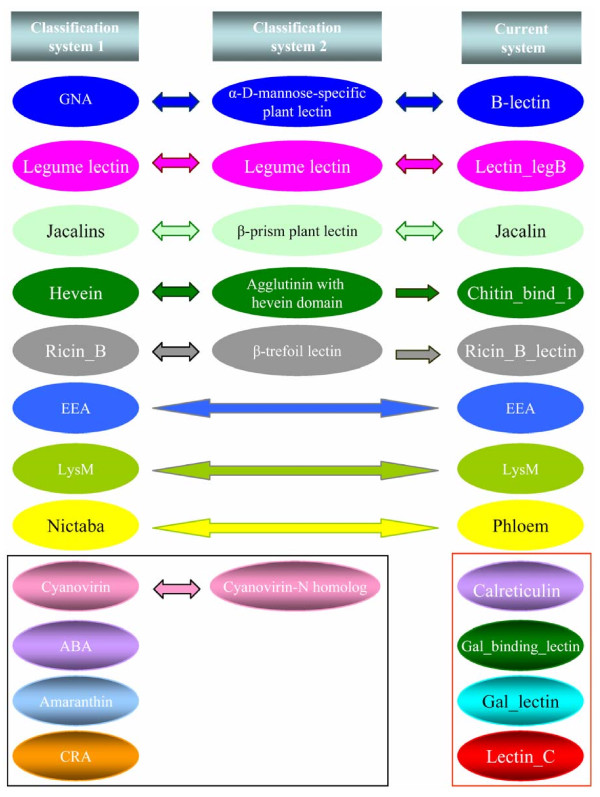
**Comparison of the new classification system with two other systems**. Classification system 1 is based on the description by Van Damme et al., (2008) [7]. The system 2 was based on 3D structures of lectins as described in the website: http://www.cermav.cnrs.fr/lectines/. The lectin families with no detectable member in soybean, rice and *Arabidopsis *were indicated by the rectangle with black lines and newly identified lectin families in the new classification system were framed by the rectangle with red lines.

Based on the phylogenetic analyses of all genome-widely identified lectin genes in three organisms, we have proposed a new classification system. In this new system, each family contains members from a single carbohydrate binding-related domain. Compared with the classification system 1 [7], 8 families, including B-lectin, Lectin_legB, Jacalin, Chitin_bind_1, Ricin_B_Lectin, EEA, LysM and Phloem, have been found to match their corresponding families each other. Their coordinates are GNA, Legume lectin, Jacalins, Hevein, Ricin_B, EEA, LysM and Nitaba lectin families, respectively (Figure 2). No lectin member was identified in both rice and *Arabidopsis *genomes that was classified into the remaining 4 families including Cyanovirin, ABA, Amaranthin and CRA, which were reported by Van Damme et al (2008) [7]. The result was confirmed by our searches and the soybean genome may also lack lectins from these families (Figure 2). Thus, these families may be species-specific.

On the other hand, we also compared our new system with the classification system 2 as described in the website http://www.cermav.cnrs.fr/lectines/. Among the 6 classes of lectins in this system, 5 of them including α-D-mannose-specific plant lectin, Legume lectin, β-prism plant lectin, Agglutinin with hevein domain and β-trefoil lectin have been detected to match their corresponding families including B-lectin, Lectin_legB, Jacalin, Chitin_bind_1 and Ricin_B_lectin classes in our new system (Figure 2). Similarly, the class "Cyanovirin-N homolog" members have not been detected in our new system since they are mainly from fungi and bacteria. Furthermore, based on our taxonomic coverage analyses by the InterPro database [63], these 12 families are ubiquitous in higher plants while only limited species could be detected with the four families identified by the system 1 including Cyanovirin, ABA, Amaranthin and CRA. Thus, our new system can be used for the general lectin classification in higher plants and can not be used for species-specific lectins.

### Genome-wide identification reveals new classes of lectin families in soybean, rice and *Arabidopsis*

Interestingly, we have detected four more families including Calreticulin, Gal_binding_lectin, Gal_lectin and Lectin_C, which are not detected in the two classification systems (Figure 2). They were usually detected in animals. However, the Calreticulin, Gal_binding_lectin and Gal_lectin families were also previously identified as putative plant lectins [64]. To our knowledge, no experimental data has been reported to confirm these members as plant lectins. For the Calreticulin family, many members have been identified in plants [65]. However, no evidence shows their lectin activities although the family has been regarded as one of animal lectin groups [66]. The Lectin_C family is known as S-type lectins in animals and fungi. We have detected 2 members of the lectin_C family lectins in soybean and one member in both rice and *Arabidopsis *(Figure 1). Expression evidence is from EST for 2 soybean members and from full-length cDNAs for rice and *Arabidopsis *members, suggesting their presence in plants. Moreover, domain amino acid sequence alignments showed that these 4 Lectin_C domains from plants have at least 30% sequence identities when compared with the most closely related domains from animals, demonstrating their possible function as C-type lectins.

**Figure 3:**
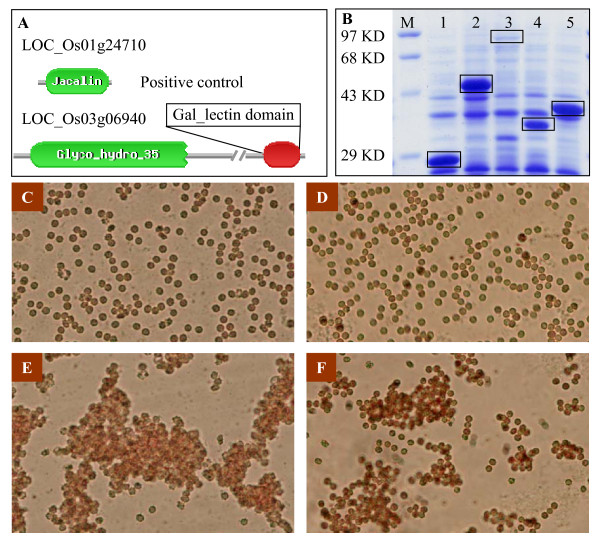
**Hemagglutination activity assay of the rice Gal_lectin member *LOC_Os03g06940***. (A) Domain structures of the positive control (up) and the Gal-lectin member detected by Pfam database. (B) *In vitro *expression of the recombinant proteins as shown by rectangles. M, molecular marker; 1, expressed GST tag in the vector pGEX-6p as a negative control; 2, recombinant protein from the Gal_lectin domain and its flanking region; 3, from the Glyco_hydro_35 domain; 4, exactly from the Gal_lectin domain; 5, from the Jacalin domain as a positive control. (C) and (D) No agglutination of the erythrocytes was observed when *E. coli *extracts from the empty vector (lane1 in B) or from the recombinant construct containing the Glyco_hydro_35 domain (lane 3 in B) were used for the activity assay. (E) and (F) Agglutination of erythrocytes by the *E. coli *extract from positive control (E) or from the recombinant construct containing the Gal_lectin domain (F). Recombinant proteins from both lane 2 and 4 show the agglutination activity and only the latter is shown in (F).

Both the Gal_binding_lectin and Gal_lectin families are known as S-type lectins. For these two families, expression evidence was from EST for most of soybean members and from full-length cDNAs for both rice and *Arabidopsis*. The fact demonstrates the presence of these two domains in plants. However, their domain amino acid sequences share low identities (around 30%) when compared with the most closely related domains from animals. Thus, their functions as lectins should be demonstrated by testing their activities. We have randomly selected one of these newly identified lectin members for further analyses. This gene was named as *LOC_Os03g06940 *and contains two domains including both Glyco_hydro_35 and Gal_lectin (Figure 3A). The cDNA sequences from different domain regions were cloned into the expression vector pGEX-6p. The GST fusion proteins were expressed in the *Escherichia coli *BL21 (Figure 3B). The recombinant protein extracts were used for detecting lectin activities by hemagglutination testing (Figure 3C-F). This method has been widely used for testing the lectin activity [55,67]. Our data show that the Gal_lectin domain indeed exhibits the agglutination activity whereas the Glyco_hydro_35 domain has no this function. The data suggest that this should be a new lectin identified in plants although its carbohydrate-binding specificity is not yet determined.

On the other hand, some of these four families of newly identified lectins contain other domains. Thus, many of them may be annotated as other proteins. For example, most of Gal_lectin or Gal-Binding lectin family members have been annotated as β-galactosidase or galactosyltransferase since these members contain Glyco_hydro_35 or Galactosyl_T domain, respectively. These domain combinations are also observed in bacteria, nematodes and higher animals. Therefore, they can be regarded as chimerolectins if their sugar binding properties can be experimentally validated.

### Different families show difference in their expansion and large-scale expansions occurred after the divergence from their ancestors

**Figure 4:**
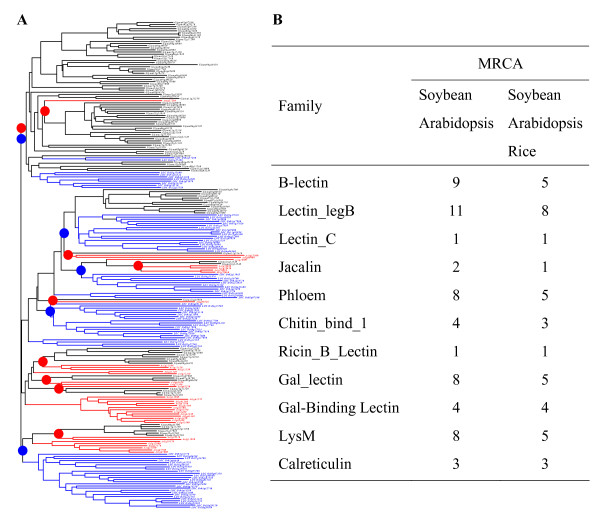
**Phylogenetic analysis and evolutionary dynamics of the lectin superfamily**. (A) Phylogenetic analysis of the B-lectin family members in Soybean, rice and *Arabidopsis*. B-lectin domain amino acid sequences were employed to construct phylogenetic tree using the bootstrap method with a heuristic search of the PAUP 4.0b8 program. Generated trees were similar to those from Bayesian analyses. Ancestral units were defined according to Shiu et al (2004) [68]. Red circles represent the most recent common ancestor (MRCA) units between soybean and *Arabidopsis *and black circles indicate the MRCA units among soybean, *Arabidopsis *and rice. (B) The MRCA analyses in all 12 lectin families.

Different lectin families exhibited differential expansions and as a result, plants have evolved into different sizes of lectin families. To infer the patterns of gene family expansions, we aligned the domain sequences of all members of each family from three organisms. The alignments were used to generate the phylogenetic trees and one of them is shown in Figure 4A, which was constructed with B-lectin domain sequences. The phylogenetic tree was then broken down into ancestral units, which were clades that were present before the divergence of these organisms according to the method described by Shiu et al (2004) [68]. The nodes of the most recent common ancestor (MRCA) were labeled with solid red circles between soybean and *Arabidopsis *and with black circles among 3 analyzed organisms (Figure 4A). We found that there were 9 ancestral units among 105 soybean and 38 *Arabidopsis *B-lectin members; however, only 5 MRCAs have been detected among total of 230 B-lectin family members from these three organisms (Figure 4A). The result indicated that the ancestral organism contained small family of B-lectin members and suggested that the large scale of expansions occurred after the divergence from their ancestors. A similar method was used for searching the lectin members of the ancestral organism for the remaining 8 families of lectins. The analysis showed that the ancestral organism between two dicot plants or among these three organisms also contained small numbers of lectin families (Figure 4B). Furthermore, we have also found several species-specific sub-family members for all three organisms (Figure 4A). All these results confirmed that the large scale of expansions occurred after the divergence from their common ancestors.

### Tandem and segmental duplications represent the major mechanism of lectin family expansion

Based on the genome-scale duplication data in *Arabidopsis *and rice [69,70], one ancestral gene in the MRCA of *Arabidopsis *and rice may give birth to four and two novel genes in *Arabidopsis *and rice, respectively [71]. Thus, maximal 20 *Arabidopsis *and 10 rice B-lectin genes could be born on the basis of 5 members in the MRCA between these two species. However, they have much more numbers of lectin genes, suggesting that other mechanisms, such as tandem, segmental duplication and/or retroposition must have also contributed to the expansion of the lectin gene super family.

**Figure 5:**
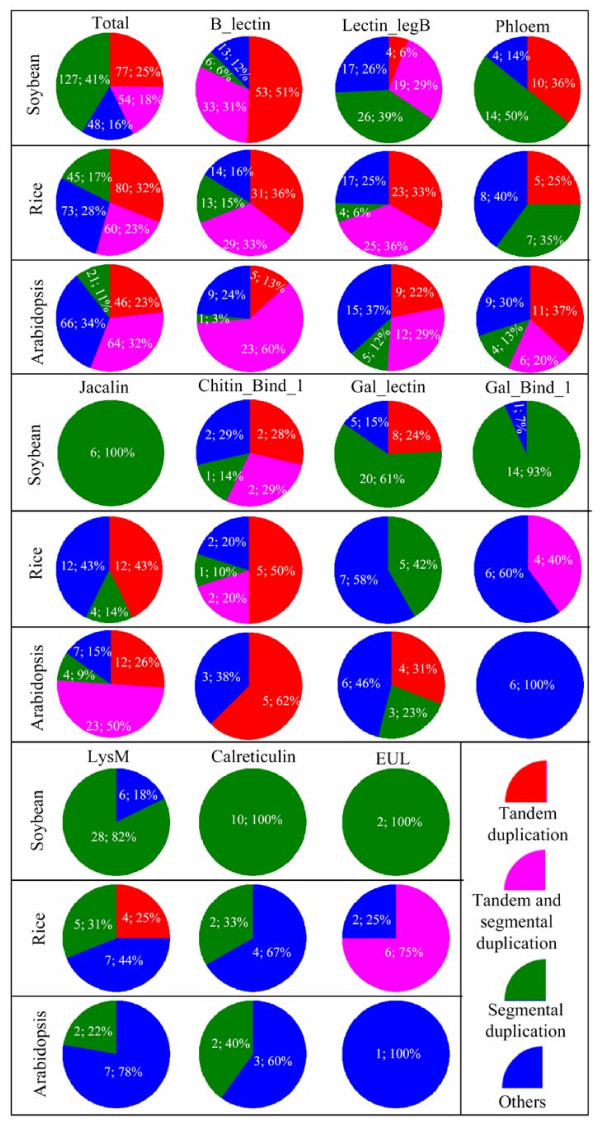
**Expansion mechanisms of lectin genes**. Pie diagrams were given to indicate the contributions of tandem (red), segmental (green) duplications and both of them (pink) as well as other mechanisms (blue) to the expansion of lectin superfamily in soybean, *Arabidopsis *and rice genomes. In each pie diagram, the number prior to the semicolon ";" indicates the total expanded members by different expansion mechanisms and its percentage is given following the semicolon.

To investigate the contribution of tandem duplication to the expansion of lectin genes, we examined the chromosomal distribution of members in each family of all three organisms. Such analyses show that many genes are clustered together according to the criterion as described in Methods section, suggesting that they were the results of tandem duplication. Totally, we have detected 131 (43%), 140 (55%) and 110 (551%) lectin genes being involving in tandemly duplicated events in soybean, rice and *Arabidopsis*, respectively (Figure 5). On the other hand, we also genome-widely identified segmental duplications in soybean, rice and *Arabidopsis *genomes and then searched the duplication blocks that contain lectin genes. In this case, tandemly arrayed genes were treated as a single gene copy. We have detected total of 71 segmental blocks and their duplicated blocks with the same family of lectin genes in soybean (Additional file 4_sheet1). These genes were from total of 11 different families. One of the families is the Lectin_C and only one member was detected in both rice and *Arabidopsis*. However, two members were detected in soybean, which was due to the segmental duplication as shown in the Additional file 4_sheet1. Similarly, we have 19 and 15 segmental and their duplicated blocks with lectin genes in rice and *Arabidopsis*, respectively (Additional file 4_sheet 2 and 3). In summary, we have detected total of 181 (59%), 105 (40%) and 85 (43%) lectin genes being involving in segmentally duplicated events in soybean, rice and *Arabidopsis*, respectively (Figure 5). These data suggested that both tandem and segmental duplications play a major role in lectin gene expansions. Further investigation showed that some tandemly duplicated genes were also within segmentally duplicated regions with overlaps between these two duplications. Figure 5 summarizes the contributions of tandem and segmental duplications to the expansions of various lectin gene families in three different organisms. Generally, the highest contribution rate was observed in soybean with up to 84% of lectin genes were involved in tandem and/or segmental duplications and the remaining 16% of lectin genes were from other mechanisms. In both rice and *Arabidopsis*, similar results were observed with around 28% and 34% of lectin genes were from other mechanisms in general (Figure 5). However, the contributions of tandem and segmental duplications to lectin expansions differ among different lectin families. For example, only 12-24% of B-lectin family members were from the other expansion mechanisms while the corresponding percentage has been up to 25-37% for Lectin_legB family (Figure 5). Furthermore, a same family also exhibits differences in their expansions among three organisms. For example, 14 of 15 Gal_Bind_1 family members were within segmentally duplicated regions in soybean whereas only 4 of 10 rice members have been involved in such events and no member in *Arabidopsis *has been born by tandem/segmental duplications (Figure 5). These facts may be regarded as one of reasons contributing to differential expansions of lectin members in different families of a same genome or in different genomes in a same family.

Besides the lectin superfamily, many other gene families also expanded themselves by tandem and/or segmental duplications in Arabidopsis and rice [72-74]. Since the soybean (*Glycine max*) genome sequencing data were released recently, limited data is available on the genome-wide identification of a gene family and its expansion history. We have genome-widely analyzed segmentally duplicated chromosome blocks in soybean. We found that many gene families in soybean also expanded themselves by segmental duplication such as AP2 domain encoding genes (estimated members: 345), WRKY transcription factors (estimated members: 176) and heavy-metal-associated domain encoding genes (estimated members: 127) and so on. For example, we have detected 55 out of 127 (43%) heavy-metal-associated domain encoding genes being involved in segmental duplication.

### Species-specific expansion and rapid birth-and-death evolution of some lectin families

We have observed that a same lectin family shows difference in their expansion among three species, exhibiting species-specific expansion. For example, the soybean genome encodes 105 B-lectin genes whereas only 38 members were detected in *Arabidopsis *(Figure 1). To survey the possible mechanisms leading to species-specific expansion, we examined the contribution of tandem and/or segmental duplications to their expansion of these with at least two-fold difference in family members among 3 species including the B-lectin, Jacalin, Gal_lectin and Gal-Binding_lectin families. The investigation revealed multiple possible reasons responsible for the species-specific expansion. For example, deep expansion in the soybean B-lectin family is mainly due to the over expansion of tandem genes located on non-segmentally duplicated region (Figure 5). The *Arabidopsis *Jacalin family over-expanded themselves mainly by tandem and segmental mutual duplications (Figure 5). However, the major contribution of the species-specific expansion of the Gal_lectin and Gal_binding_1 lectin families is mainly due to the segmental duplication (Figure 5).

The evidence showed that monocot branched off from dicot plants 140-150 million years ago (MYA) [75-77]. Based on the divergence era and the estimation of the MRCA among soybean, rice and *Arabidopsis *as shown in Figure 4, the birth rate of B-lectin genes (counting only survival copies) is at least 13.3, 10.9 and 4.4 genes per 100 million years (MY) per ancestral gene in lineages leading to soybean, rice and *Arabidopsis*, respectively. The average rate of gene duplication is around 1 gene per 100 MY per ancestral gene in eukaryotes such as *Homo sapiens*, *Mus musculus*, *Drosophila melanogaster*, *Caenorhabditis elegans*, *Saccharomyces cerevisiae *and *Arabidopsis thaliana *[78]. Thus, these rates are significantly higher than the average one. Similarly, we have estimated the birth rates in other expanded families (Additional file 5). The analyses show that different families exhibit difference in their gene duplication rates. The highest rate was observed in the Jacalin family, where 30 genes per 100 million years in the lineage leading to *Arabidopsis*. The lowest rate was 0.3, significantly lower than the average rate, and occurred in the *Arabidopsis *Gal-Bind_lectin and Calreticulin families. On the contrary, by comparing the expansion mechanisms (Figure 5) and gene birth rates (Additional file 5), we found that high gene birth rates are mainly due to tandem and/or segmental duplications. For these families, whose members being involving in both tandem and segmental duplications account for more than 50% of total identified lectins in all families and species, their gene birth rates were estimated at least 2 genes per 100 MY per ancestral gene. In addition, the rates of gene birth in multiple lectin gene families may be under-estimated. The actual numbers of duplicate genes that once existed in three species could be more than that we have detected as some duplicate copies might have already been deleted from the genome or been evolved into pseudogenes that are not included in this study. We have detected 86, 101 and 20 members from total of 12 families with partial domain sequences in soybean, rice and *Arabidopsis*, respectively. These partial sequences usually lack regions corresponding to sugar binding properties. Thus, they may be nonfunctional and could be regarded as pseudogenes. There seems to be a correlation between tandem duplication and pseudogenization since most of these members were from tandem duplication related expansion. On the other hand, some duplicated copies might be too divergent to be recognized now, which may also contribute to the under-estimation. Thus, higher rates of gene duplication should have occurred for some lectin families in these three species.

### Differential expansion patterns of tandem and segmental dual duplications among three organisms

**Figure 6:**
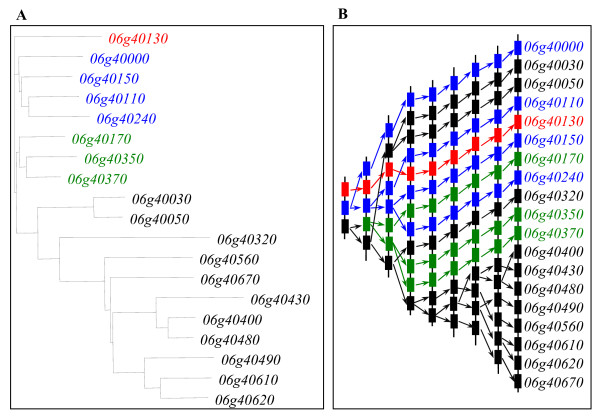
**Expansion of one of the largest tandam clusters in B_lectin family by tandem duplications in soybean**. (A) Phylogenetic relationships of 19 members from the tandem cluster. (B) Hypothetical origins of tandemly duplicated genes. The prefix "Glyma" of gene locus names have been omitted for convenience.

Since the tandem duplication has been observed to largely contribute to the birth of new lectin genes, we further analyzed their expansion patterns. Domain amino acid sequences from the same tandem cluster were aligned first and were then used for the construction of their phylogentic tree, which were employed to analyze their expansion history. Based on such analyses, we found that parental genes were not always physically linked to their descendant genes and different expansion rates were observed for tandem genes in a same array. One of such examples was shown in Figure 6. In this example, we analyzed the patterns of tandem duplication in one of the largest tandem clusters in the soybean B-lectin family. We first investigated their expansion history by phylogenetic analyses (Figure 6A). The 19 tandem genes are in three clades, suggesting that this cluster was the results of three ancestral units, which may be evolved from ancient tandem duplication events. One of them contains only one gene, i.e. *Glyma06g40130*. No expansion was detected for this gene or its expanded genes have been lost during long evolutionary history. The second clade has 4 members whereas the third clade contains 14 members. On the basis of the phylogenetic tree, we deduced the hypothetical origins of 19 genes by tandem duplication (Figure 6B). The analyses showed that these genes were generated by at least 8 rounds of tandem duplication. After expansion, these genes were then not always inserted into the loci according to their physical orders. For example, the putative tandem pair of the *Glyma06g40400 *gene is *Glyma06g40480 *but not its physical neighbor *Glyma06g40430*. On the other hand, we found that most of tandem duplication occurred by a one-gene mode, i.e. only one gene was duplicated in a one tandem duplication event as shown in Figure 6. However, these cases were also observed that two or more genes in a cluster were duplicated through a single tandem duplication event (data not shown).

**Figure 7:**
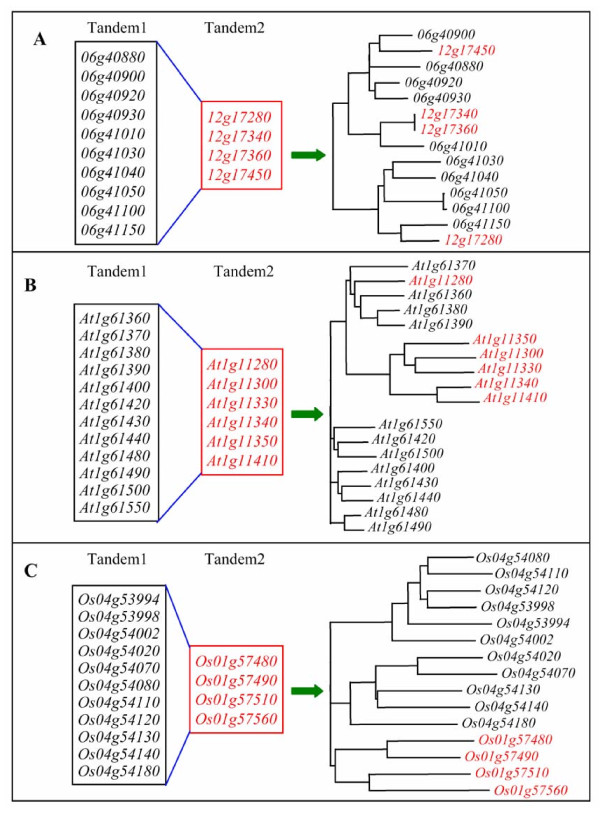
**Differential expansion of lectin genes by both tandem and segmental duplications among soybean, *Arabidopsis *and rice**. (A) and (B) Tandem-segment mixed duplication model in soybean and *Arabidopsis*, respectively. (C) Tandem-segment separated duplication model in rice. All analyzed example genes were from B-lectin family.

Based on our analyses on the expansion of the lectin superfamily by tandem and segmental duplications in three organisms (Figure 5), the evolution of considerable genes are related to both tandem and segmental duplications. To further investigate their contribution and patterns to the expansion of lectin members, segmental block pairs with at least 3 detectable tandemly arrayed genes were selected for more detail analyses. Totally, 6 pairs of segmental blocks with such criteria were detected. Three of them were from soybean including 2 B-lectin and 1 Lectin_legB pairs. Two of them were from *Arabidopsis *B-lectin and Jacalin families, respectively. The remaining one pair was from rice B-lectin family. Total of 6 phylogenetic trees were constructed for 6 pairs of segmentally and tandemly duplicated members. In most cases (5 of 6 pairs), a part of genes from the first tandem array are clustered together with another part of genes from the secondary tandem array. They exhibit tandem-segment mixed duplication model. These cases were observed in both dicot plants soybean and *Arabidopsis *as shown in Figure 7A and 7B. Such model of duplication may occur in two ways. One of them is tandem-segment-tandem duplication. In this case, the expansion was by tandemly duplication first and was then by segmental duplication followed by tandem duplication. The second way is segment-tandem-segment duplication. However, in the monocot rice genome, lectin expansion was through another model as shown in Figure 7C. In this model, genes from a tandem array were clustered together and no clade was detected with members from both tandem arrays, exhibiting a tandem-segment separated duplication model. Based on the phylogenetic relationship of these tandemly and segmentally duplicated members, one reasonable explain is that they underwent segmental duplication first followed by tandem duplication.

### Limited but significant contribution of retrogenes to the birth of new lectin genes in both soybean and rice

**Figure 8:**
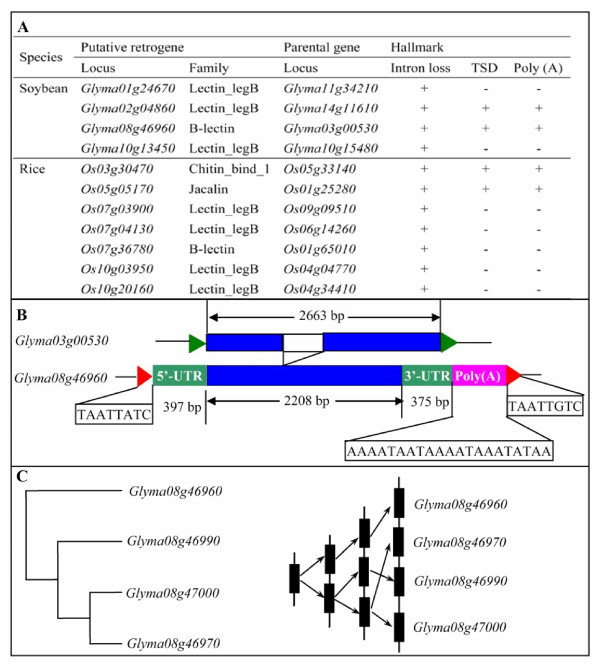
**Retrogenes and their contributions to the expansion of lectin genes**. (A) Retrogenes identified in soybean and rice genomes. (B) An example showing evolution of a lectin gene *Glyma08g46960 *by retrotranspositions from another lectin gene *Glyma03g00530*. The red arrowheads indicate the detected flanking direct repeats in an identified retrogene. The green arrowheads show the start (left) and end (right) positions of an annotated gene. The green boxes indicate the predicted 5'-UTR (left) and 3'-UTR (right) in a retrogene. The blue boxes indicate exons in both retrogenes and their host genes. The white boxes indicate an intron in the parental gene. (C) A retrogene mediated tandem duplication.

Since tandem and segmental duplications can not explain all the duplication events, we expect some of other mechanisms may also contribute to the expansion of the lectin superfamily. These include both retrotransposon and transposon mediated gene expansion, which has been reported in multiple species including rice and *Arabidopsis *[79-83]. By retrotransposition, a reverse-transcribed mRNA was inserted into a new genomic position to form a retrogene. Such a retrogene is usually devoid of introns and with the presence of target site duplications and/or a poly (A) tract [82,84]. To survey the contribution of retrogenes to the expansion of the lectin superfamily, one-exon-containing lectin genes were used as query protein sequences for BLASTP searches against all two or more exon-containing lectins (Methods). The searches produced many candidate retrogenes in soybean and rice. We then manually identified these candidates by the presence of target site duplications (TSD) and/or a poly (A) tract. The analyses revealed 4 putative retrogenes in soybean and 7 in rice (Figure 8A). Some of them are still with 3 hallmarks of retrogenes. One example is the soybean retrogene *Glyma08g46960 *with both hallmarks including TSD and poly (A) tract; its parental gene *Glyma03g00530 *contains two exons (Figure 8B). On the other hand, not all identified retrogenes possess all three features, especially the poly (A) tract and short direct repeats [82,84]. These two hallmarks may no longer be recognized since they can be easily masked by base substitutions, insertions and/or deletions during a long evolutionary history [82]. Although very limited contribution has been detected by retrogenes (Figure 8A), they may play significant roles in the expansion of lectin genes. After the birth of a retrogene, it may further be expanded by tandem duplication. For example, the putative retrogene *Glyma08g46960 *was detected with tandem duplication followed by the birth of three new lectin genes including *Glyma08g46970*, *Glyma08g46990 *and *Glyma08g47000 *(Figure 8C). Moreover, no retrogene has been detected in the *Arabidopsis *lectin superfamily.

On the other hand, to investigate the contribution of DNA transposons to the expansion of lectin genes, a 100-110 Kb region including 50 Kb upstream and downstream sequences for each gene was achieved and was then used for the identification of major DNA transposons. We have analyzed class I mobile elements including *Mutator*-like transposable element (*MULE*), *CACTA *and *hAT *as well as class II *Helitron *elements presented in these regions (Methods). In *Arabidopsis*, no above mentioned DNA transposon was detected to cover the lectin gene regions. However, we have identified considerable numbers of transposons presented in the 100-110 Kb regions in soybean. For example, 14 *MULE*-like, 3 *CACTA *and 20 *Helitron *elements have been detected and no *hAT *element has been identified in soybean. However, most of these elements are located outside the corresponding lectin genes. Only 4 genes were identified within 4 *Helitron *elements. These genes include *Glyma08g07040 *(Lectin_legB), *Glyma09g21970 *(Gal_lectin), *Glyma13g42210 *(Chitin_bind_1) and *Glyma20g02580 *(Phloem). They are candidates to contribute to the expansion of lectin genes. Thus, DNA transposon elements have limited contribution to the expansion of the lectin superfamily in soybean. A similar situation was also observed in rice. In this organism, we have detected 6 *MULE*-like elements and all of them are located within lectin genes. Five of these elements are within introns of corresponding lectin genes including *LOC_Os04g03579 *(Lectin_legB), *LOC_Os04g34410 *(B_lectin), *LOC_Os05g35360 *(Gal_Lectin), *LOC_Os11g39420 *(Jacalin) and *LOC_Os12g24170 *(Gal_Lectin). The remaining one contributes the insertion of 6 exons of the lectin gene *LOC_Os10g18400 *(Gal_Lectin) and this *MULE*-like element was named TI0006870 as described by Juretic et al (2005) [80]. In addition, the remaining elements including *CACTA*, *hAT *and *Helitron *have not been detected to contribute to the expansion of the lectin superfamily in rice.

### Expression profiling of the lectin superfamily in soybean, rice and *Arabidopsis*

**Figure 9:**
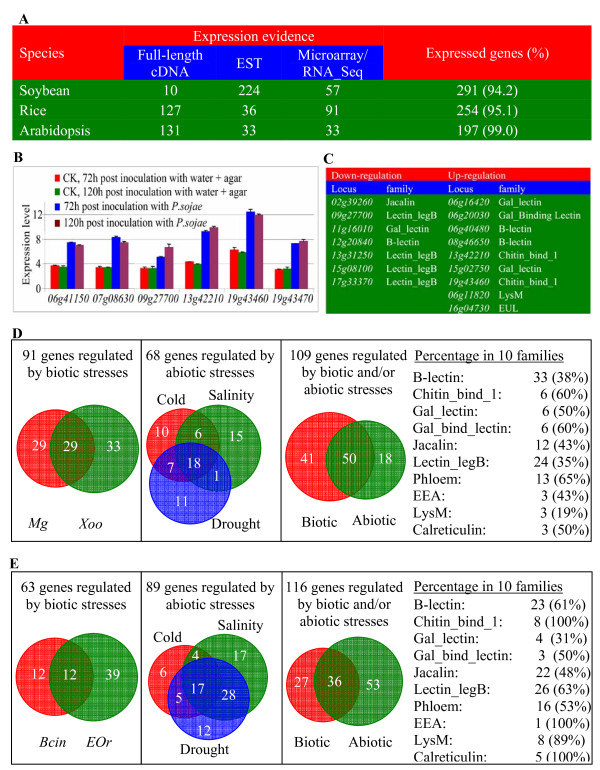
**Expression profiles of lectin superfamilies in soybean, rice and *Arabidopsis***. (A) A general expression analysis showing the total expressed lectin genes in three organisms. (B) Five lectin genes showing up-regulation by the pathogen *P. Sojae *in soybean. The prefix "*Glyma*" in locus names was omitted for convenience. (C) Seven down-regulated and six up-regulated lectin genes by soybean cyst nematode. (D) and (E) Transcriptional profiles of lectin genes under various biotic and abiotic stresses rice and *Arabidopsis*, respectively.

To survey the expression of all identified lectin genes, expression evidence was obtained from various expression databases as described in the Methods section. We identified a gene to be an expressed gene if a full-length cDNA, EST and/or microarry/RNA_Seq [85] could be available. Based on our investigation, more than 90% of lectin genes from all three organisms are regarded as expressed genes (Figure 9A). For soybean, only 10 lectin genes were experimentally detected to contain full-length cDNAs and most expression evidence was from ESTs. In contrast, 127 of 267 (48%) lectin genes have full-length cDNAs as expression evidence in rice and more than half of *Arabidopsis *lectin genes (131 of 199 or 66%) have their corresponding full-length cDNAs.

To investigate the expression profiles under various biotic and abiotic stresses, microarray/MPSS data were achieved from various databases and statistic analyses were carried out (see Methods) to determine if the expression of a lectin gene is regulated by biotic/abiotic stresses. For soybean, only 71 lectin genes were probed in the Affymetrix microarray chips based on the annotation http://soybase.org/AffyChip/index.php. These genes are from 9 of 12 lectin families including B_lectin, Lectin_legB, Jacalin, Lectin_C, Chitin_bind_1, Gal_lectin, Gal_binding_Lectin, EEA and LysM. To analyze the expression profiles of these probed genes, microarray data were downloaded from Gene Expression Omnibus (GEO) DataSets [86] and Arrayexpress database [87] as described in the Methods section. We have analyzed the effects of both *Phytophthora sojae *and cyst nematode infections on the lectin gene expression. The analyses showed that 6 lectin genes exhibited up-regulation after the infection of the pathogen *P. sojae *(Figure 9B). These lectin genes include *Glyma06g41150 *(B-lectin), *Glyma07g08630 *(LysM), *Glyma09g27700 *(Lectin_legB) and three Chitin_bind_1 family genes: *Glyma13g42210*, *Glyma19g43460 *and *Glyma19g43470*. On the other hand, we have detected 16 lectin genes with changed expression by cyst nematode including 7 down-regulated and 9 up-regulated genes (Figure 9C). Among them, three genes were co-regulated by both biotic stresses including *Glyma09g27700*, *Glyma13g42210 *and *Glyma19g43460 *(Figure 9A and 9B). Thus, total of 19 out of 71 lectin genes (27%) have been detected to be involved in biotic stress-related signaling pathways.

On the other hand, we are also very interested in the soybean lectin genes related to legume-rhizobium symbiosis. We have found numbers of soybean-specific lectin family members based on the phylogenetic analysis. We are wondering whether some families of lectins involved in legume-rhizobium symbiosis are unique to soybean or are absent/non-functional in rice and Arabidopsis. A 36, 760 probe-containing microarray analysis showed that at least 73 lectin-related probes have been detected with *Bradyrhizobium japonicum*-regulated expression patterns [88]. These probes were from both soybean-specific and non-specific lectin genes. No enough evidence shows that soybean has evolved into some soybean-specific lectins specially for the legume-rhizobium symbiosis. For example, the Lectin_legB family member *Glyma02g18090 *was up-regulated by the *rhizobium*. However, it is not soybean-specific lectin gene. Generally, our data have demonstrated that soybean lectin families have been involved in legume-rhizobium symbiosis.

In rice and *Arabidopsis*, expression data of most lectin genes are available under various biotic and abiotic stresses. We have downloaded all expression data of rice lectin genes under biotic and abiotic stresses from rice MPSS database (Methods). Data analyses showed that total of 58 and 62 lectin genes were differentially expressed after infection by the fungus and bacterium pathogens *Megnaporthe grisea *(*Mg*) and *Xanthomonas oryzae *pv *oryzae *(*Xoo*), respectively, among which 29 genes were regulated by both pathogens (Figure 9D, Additional file 6). Thus, the expression abundance of total of 91 genes (34%) was regulated by biotic stresses (Figure 9D). On the other hand, we have detected 68 genes (25%) with differential expression under cold, drought and/or high salinity stresses, among which 18 genes were regulated by all of three abiotic stresses (Figure 9D, Additional file 6). Totally, we have detected 109 of 267 rice lectin genes (41%) with differential abundance under biotic and/or abiotic stresses as 50 genes were regulated by both biotic and abiotic stresses (Figure 9D). The percentages of regulated genes in each family vary from 19% for the LysM to 65% for the Phloem families (Figure 9D).

In *Arabidopsis*, 63 of 199 lectin genes exhibited difference in their transcript abundance after the infection by the fungus and bacterium pathogens *Botrytis cinerea *(*Bcin*) or *Erysiphe orontii *(*EOr*), accounting for 32% of the lectin superfamily members (Figure 9E, Additional file 7). Up to 89 genes (45%) were regulated by cold, drought and/or high salinity stresses and this ratio is significantly higher than that in rice (Figure 9E, Additional file 7). Thus, total of 116 genes (58%) underwent biotic/abiotic-stress regulation in their expression and each family showed difference in its response to both stresses with the percentages ranging from 31% for the Gal_lectin to 100% for the Chitin_bind_1, EEA and Calreticulin families (Figure 9E).

Besides the expression analyses under various stresses, we have also investigated the transcript profiling among different tissues to examine if the lectin superfamily has been involved in tissue specificity and functionality. In rice, we have examined the expression abundance in 13 different tissues. Our analysis showed that 7 out of 12 lectin families have been detected to contain genes with tissue-specific expression. The percentages of tissue-specific genes are 8.0%, 40.0%, 14.3%, 41.7%, 21.4%, 4.3% and 10.0% in the B-lectin, Chitin_binding_1, EEA, Gal_Lectin, Jacalin, Lectin_legB and Phloem families, respectively (Additional file 8). These lectin genes were preferentially expressed only in one or two tissues. In Arabidopsis, the transcript abundance was investigated among six different tissues including callus, germinating seedlings, inflorescence (mixed stages), leaves (21 day), root (21 day), silique (24-48 hour post-fertilization). Based on our analysis, only 4 out 12 lectin families contain genes with tissue-specific expression, significantly less than rice. These genes were from the B-lectin, Jacalin, Lectin_legB and Phloem families. The percentages of tissue-specific genes in these 4 families are 23.7%, 2.2%, 9.8% and 16.7%, respectively (Additional file 9). These data demonstrated that lectin genes not only play a role in specific stress conditions some lectin families but also have been involved in tissue specific biological functions.

### Expression divergence among tandemly and segmentally duplicated genes

Our data show that both tandem and segmental duplications have significantly contributed to the expansion of the lectin superfamily and high percentage of lectin members exhibited differential expression patterns under various biotic and abiotic stresses. To explore the effect of both tandem and segmental duplication on the expression patterns, we have carried out a detail analyses on their expression divergence among tandemly or segmentally duplicated members.

**Figure 10:**
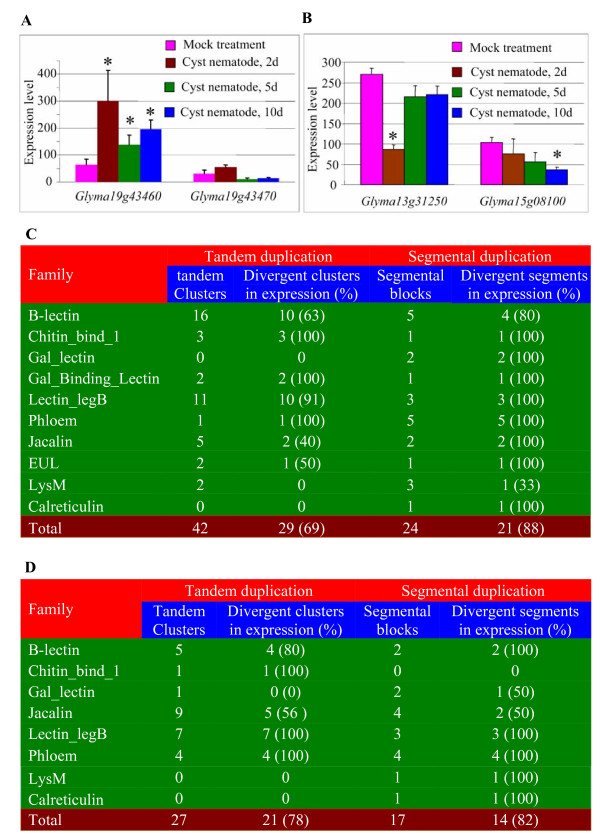
**Expression divergence among tandemly and segmentally duplicated lectin genes in soybean, rice and *Arabidopsis***. (A) and (B) Examples of expression divergence under cyst nematode treatment after tandem (A) or segmental (B) duplications in soybean. (C) and (D) The effect of tandem and segmental duplications on gene expression divergence of different lectin families under abiotic and biotic stresses in rice and *Arabidopsis*, respectively.

In soybean, among analyzed 71 lectin genes, four members were from two tandem clusters. One of them exhibits expression divergence after infection by cyst nematode as shown in Figure 10A. On the other hand, we have also detected 8 segmentally duplicated blocks among 71 probed lectin genes in Affymetrix chips. Three blocks (38%) exhibits expression divergence after infection by *P. sojae *or cyst nematode. For example, the gene *Glyma12g17280 *exhibits no difference in its expression after infection by *P. sojae *whereas its segmentally duplicated coordinate *Glyma06g41150 *was regulated by the pathogen (Figure 10B). Similar case was observed in another segmentally duplicated coordinates *Glyma04g34620 *and *Glyma06g20030 *(up-regulated by cyst nematode as shown in Figure 9C). However, for the third coordinates *Glyma13g31250 *and *Glyma15g08100*, both of them were down-regulated by cyst nematode and they exhibited difference in response to the infection time (Figure 10B).

In rice, we have analyzed the expression divergence of total of 42 tandem clusters and 24 segmental blocks. If the expression patterns of any members of a tandem cluster exhibit difference from any other members in the cluster under either any biotic or abiotic stresses, this cluster is regarded as a divergent cluster in their expression. A similar criterion has also been applied to the evaluation of expression divergence in segmental duplications. Based on the evaluation, we have identified 29 tandem clusters and 21 segmental blocks with differential expression under various biotic and abiotic stresses, accounting for 69% of total clusters and 88% of total segmental blocks, respectively (Figure 10C). Further analyses show that different lectin families exhibit significant difference in their expression divergence after tandem duplication, ranging from 40% for the Jacalin family to 100% for the Chitin_bind_1, Gal_binding and Phloem families (Figure 10C). On the other hand, for segmentally duplicated blocks, most of them show expression divergence for most of families (Figure 10C). However, the percentage may be over-estimated since the expression divergence from tandem duplication were also included if a tandem duplication occurred after segmental duplication.

In *Arabidopsis*, We have investigated total of 27 tandem clusters and 17 segmental blocks. Around 78% of tandem clusters and 82% of segmental blocks have been observed with regulated expression patterns under either biotic or abiotic stresses (Figure 10D). In summary, expression data from soybean, rice and *Arabidopsis *demonstrate that tandem and segmental duplications significantly contribute to gene expression divergence under various biotic and abiotic stresses.

### Domain combinations, high percentages of expression divergence and biological functions of lectin genes

Due to the presence of hololectins and chimerolectins, we have submitted all lectin protein sequences for other domain detection. The analyses revealed that many lectins also contained other domains besides the carbohydrate-binding domain. We have detected at least 5 other domains presented in at least 30% of corresponding family members. They are Kinase domain for B-lectin, Lectin_legB and Lectin_C families, F-box domain for phloem family, Glyco_hydro_19 domain for chitin_bind_1 family, Glyco_hydro_35 domain for Gal_lectin family and Galactosyl_T domain for Gal-Binding Lectin family (Table 1). Among them, more detail analyses have been carried out for the F-box-containing phloem lectins and they may play a role in nucleocytoplasmic protein degradation [60,89]. The presence of other domain suggests the more complicated functions and evolutions of plant lectins.

**Table 1:** Taxonomic coverage and domain combination of the lectin superfamily

		**Other domain**^**a**^			
		
			Percentage (%)		
			
Family	Taxonomic coverage	Name	Soybean	Rice	*Arabidopsis*
B-lectin	Mainly in plants	Kinase	90.5	92.0	78.9
Lectin_legB	Mainly in plants		72.7	87.0	85.4
Lectin_C	Mainly in animals		100.0	100.0	100.0

Jacalin	Plants, animals and microorganisms	-	-	-	-

Phloem	Only in plants	F-box	82.1	46.4	56.7

Chitin_bind_1	Mainly in plants and fungi	Glyco_hydro_19	71.4	80.0	87.5

Ricin_B_Lectin	Plants, animals and microorganisms	-	-	-	-

Gal_lectin	Mainly in animals	Glyco_hydro_35	100.0	100.0	69.2

Gal-Binding Lectin	Mainly in animals	Galactosyl_T	100.0	100.0	100.0

Calreticulin	Plants and animals	-	-	-	-

EEA	Mainly in plants	-	-	-	-

LysM	Plants, animals and microorganisms	-	-	-	-

^a ^Only domains presented in at least 30% of corresponding family members were listed

One may concern why the lectin families have evolved in these ways and how these duplicated genes are retained and whether there are any biological needs or advantages to drive their evolution. Domain combinations are the processes that generate new genes and functional divergences [90,91]. In this study, we have detected 5 domains presented in 7 families (Table 1). One of them is the kinase domain, which was presented in three families including B-lectin, Lectin_legB and Lectin_C. Here we focus on the B-lectin and Lectin_legB families since only one or two members were detected for the Lectin_C family in plants. For these two families, the combination with kinase domain did not occur in animals, bacteria and fungi, suggesting that the domain composition was established during the course of plant evolution. Phylogenetic analyses showed that most of these domains were from the receptor-like kinase (RLK)/Pelle family [68]. Interestingly, this family also underwent a rapid birth-and-death evolution in plants [68]. Thus, both lectin and kinase domains were maintained by according evolution.

We try to further elucidate the biological benefits to drive the expansion and retention of the lectin superfamily by surveying the effect of tandem and/or segmental duplications on the expression divergence under biotic and abiotic stresses. Previous reports have showed that tandemly duplicated genes tend to be involved in biotic and abiotic stresses [72,83]. Many of the RLK family members are B-lectin or Lectin_legB domain-containing proteins in rice and *Arabidopsis *[68]. Interestingly, the expression data also support the importance of this family in stress response [92]. These results further confirmed the co-evolution of B-lectin/Lectin_legB and kinase domains. Our expression data suggest a link between biotic/abiotic stresses and tandem/segmental duplications in lectin families and also suggest that not only tandem but also segmental duplications of lectin genes may be regarded as drivers for plants to adapt various environmental stresses through duplication followed by expression divergence, thus, providing an explanation for why some of lectin families exhibit large expansion. Highly divergent expression profiles also demonstrate that each member of this gene superfamily may play specialized roles in a specific stress condition and function as a regulator of various environmental factors such as cold drought and high salinity stresses as well as biotic stresses. The detection of tissue-specifically expressed genes further demonstrated the comprehensive biological functions of this superfamily.

## Conclusions

Our data show that higher plant genomes encode large numbers of lectin proteins. These proteins can be phylogenetically classified into 12 different families and four of them consist of recently identified plant lectin members. Further analyses show that some of lectin families exhibit species-specific expansion and rapid birth-and-death evolution. Tandem and segmental duplications have been regarded as the major mechanisms for lectin expansion. Our analyses also shows that lectin genes have been involved in biotic/abiotic stress regulations and tandem/segmental duplications may be regarded as drivers for plants to adapt various environmental stresses through duplication followed by expression divergence. All in all, our studies provide a new outline of the plant lectin gene superfamily and advance the understanding of plant lectin genes in their evolution, expansion and biotic/abiotic stress-related biological functions.

## Methods

### Databases, annotations and sequence retrieval

Three different databases have been selected to retrieve all lectin genes encoded by the soybean, rice and *Arabidopsis *genomes. The annotated protein sequences from these three genomes have been downloaded from the following websites: http://www.phytozome.net/soybean for soybean (Glyma1.0), http://rice.plantbiology.msu.edu for rice (release 6) and http://www.Arabidopsis.org for *Arabidopsis *(TAIR8).

All lectin domains were achieved from both Pfam and EBI database http://www.ebi.ac.uk/. Key sequences of various domains were obtained from the Pfam databases. However, the key sequences of the phloem and EEA lectin domains were obtained from Dinant et al (2003) [60] and Van Damme et al (2008) [7], respectively, since no Pfam ID is available in the database. These key domain sequences from each lectin domain were aligned by ClustalX 2.0 [93] and were then used to generate HMM profiles for HMM searches with e-value cutoff of 1.0. After filtration by domain confirmation using both the Pfam and the SMART databases, sequences with full-length domain were used for BLAST searches to achieve more lectin sequences.

### Sequence alignment and phylogenetic analysis

The DNASTAR program was used for the preliminary sequence manipulations. The sequence alignment was generated using ClustalX (Version 2.0) with manual adjustments using lectin domain sequences from various lectin families. The aligned amino acid sequences formed the basis for the phylogenetic analysis using the program Mac PAUP 4.0b8 (ppc) http://www.paup.csit.fsu.edu and MrBayes 3.1 [94] according to the description by Jiang and Ramachandran (2006) [95].

### Detection of duplication-related lectin genes

Tandemly duplicated lectin genes in soybean, rice and *Arabidopsis *were identified by three criteria: (1) they are less than or equal to 10 genes apart; (2) they belong to the same lectin family; and (3) they are within 100 kb for *Arabidopsis *or 350 kb for both soybean and rice as suggested by Lehti-Shiu et al. (2009) [92].

The genome-wide identification of segmentally duplicated chromosome blocks has been carried out previously in soybean http://www.phytozome.net/soybean.php, rice [70,73] and *Arabidopsis *[96]. We examined the segmental duplicates by comparing positions of lectin genes with known duplicated chromosomal blocks. Since some of these investigations only dealt with relatively recent (for soybean) or ancient (for *Arabidopsis*) duplication events, we also compared the flanking regions (50 kb upstream and downstream) of the lectin gene pairs in these two species to identify the ancient (for soybean) or recent (for *Arabidopsis*) duplicated blocks according to the method [71].

### Detection of transposable element (TE)-related lectin genes

To detect the contribution of class I TEs (retrotransposons) to the expansion of lectin gene families, possible retrogenes in the lectin superfamily were identified. Lectin genes encoded by single exon were subjected to BLASTP searches against all the remaining lectin protein sequences with two or more exon-containing coding sequences. Homologs were collected for further analysis while minimum 70% of queried protein coding regions were aligned with an E-value threshold at 10^-8^. We then selected candidate retrogenes based on the criteria [82].

To determine the contribution of class II TEs (DNA transposons) to the expansion of this superfamily, the flanking genomic sequences of the 50 kb upstream and downstream of lectin genes were used for the identification of 4 major transposon family members. These include mutator-like transposable element (MULE), *hAT*, *CACTA *and *Helitron *families.

We identified MULE members using similar methods as described by Jiang et al (2004) [79] and Juretic et al (2005) [80]. To identify the members of *hAT *and *CACTA *DNA transposon families, we used two separate approaches including BLASTN and HMM searches as described by one of our previous reports [97].

### Processing of expression data under biotic and abiotic stresses as well as among various tissues in soybean, rice and *Arabidopsis*

We obtained the expression evidence of a lectin gene by examining the availability of a full-length cDNA, EST or expressed microarray/RNA_seq tags in a lectin gene. The investigation was carried out by searching the following databases: http://www.phytozome.net/soybean.php for soybean, http://rice.plantbiology.msu.edu/ for rice and http://www.Arabidopsis.org/ for *Arabidopsis*.

The soybean expression data under the pathogen *Phytophthora sojae *was downloaded from the GEO DataSets [86] with accession number GSE7124. The microarray data after the infection by soybean cyst nematode were obtained from the Arrayexpress database [87] with accession number E-MEXP-808 [98]. Lectin genes with at least 2-fold difference in their average expression signals under normal and stressed conditions were subjected to students'*t*-test to determine if the genes were significantly regulated by stresses. For rice, the expression data from the MPSS database [57] were used to analyze the transcriptional profiles of lectin genes under biotic and abiotic stresses as well as among different tissues. We have analyzed the effects of cold, drought and high salinity stresses as well as bacterium and fungus pathogens on the expression of lectin genes. Similar criteria have been employed to identify differentially expressed genes as described above for soybean. For *Arabidopsis*, the recently published expression data under biotic and abiotic stresses were downloaded and differentially expressed lectin genes were identify according to the description [99]. We used the Arabidopsis MPSS database [100] to investigate the tissue-specifically expressed genes.

### *In Vitro *expression of a lectin gene and hemagglutination tests

Since we have detected three new lectin families based on the genome-wide identification, one of the family members were randomly selected for the detection of the lectin activity. The cDNA sequence of the Gal_lectin family member *LOC_Os03g06940 *with corresponding full-length cDNA (accession number: AK102192) was isolated by RT-PCR using rice total RNA as template. The cDNA sequence region corresponding to its Gal_lectin domain and the remaining region were separately subcloned into *Escherichia coli *expression vector pGEX-6P (GE Healthcare). One of the Jacalin family members *LOC_Os01g24710 *with known lectin activity [55] was used as positive control. A total of 6 mL of *E. coli *culture was collected by centrifuging at 3000 rpm for 10 min at 4°C. The collection was dissolved with 1 × PBS buffer and was sonicated on ice in short bursts. The hemagglutination test was carried out by mixing 20 μl sonicated culture extract with 20 μl 2% suspension of rabbit red blood cells. Agglutination was assessed visually after 1 h at room temperature.

## Abbreviations

ABA: *Agaricus bisporus *agglutinin; CRA: chitinase-related agglutinin; EEA: *Euonymus europaeus *agglutinin; EST: expression sequence tag; EUL: Euonymus lectin; GEO: gene expression omnibus; GNA: *Galanthus nivalis *agglutinin; HMM: hidden markov model; LysM: lysin motif; MPSS: massively parallel signature sequencing; MRCA: most recent common ancestor; MULE: *Mutator*-like transposable element; TE: transposable element; TSD: target site duplications

## Authors' contributions

SR supervised the study. SYJ conceived of the study and carried out most of the work. ZM performed the hemagglutination activity assay. All authors read and approved the final manuscript.

## Supplementary Material

Additional file 1 **The lectin superfamily in soybean**. This file lists all annotated soybean lectin members. Click here for file

Additional file 2 **The lectin superfamily in rice**. This file lists all annotated rice lectin members. Click here for file

Additional file 3 **The lectin superfamily in *Arabidopsis***. This file lists all annotated *Arabidopsis *lectin members. Click here for file

Additional file 4 **Segmentally duplicated lectin genes in soybean, rice and *Arabidopsis***. This file lists all segmentally duplicated lectin members in soybean, rice and *Arabidopsis*. Click here for file

Additional file 5 **The estimation of birth rates in 7 expanded families among soybean, rice and *Arabidopsis***. This file provides the estimation of birth rates in 7 expanded families among soybean, rice and *Arabidopsis*. Click here for file

Additional file 6 **Regulated lectin genes under various biotic and abiotic stresses in rice**. This file lists the lectin genes with up/down-regulated expression patterns under various biotic and abiotic stresses in rice. Click here for file

Additional file 7 **Regulated lectin genes under various biotic and abiotic stresses in *Arabidopsis***. This file lists the lectin genes with up/down-regulated expression patterns under various biotic and abiotic stresses in *Arabidopsis*. Click here for file

Additional file 8 **Lectin genes with tissue-specific expression patterns in rice**. This file lists the lectin genes with tissue-specific expression patterns in rice. Click here for file

Additional file 9 **Lectin genes with tissue-specific expression patterns in *Arabidopsis***. This file lists the lectin genes with tissue-specific expression patterns in *Arabidopsis*. Click here for file
